# Analyzing the Usage of Shear-Wave Elastography in Evaluating Ankylosing Spondylitis: A Literature Review

**DOI:** 10.7759/cureus.92235

**Published:** 2025-09-13

**Authors:** Jimmy Wen, Romteen Sedighi, Meraj Alam, Zohaer Muttalib, Isabel Taguinod, Shannon Dwyer, Vince Thomas, Soham Kondle, Megan Kou, Foad Elahi

**Affiliations:** 1 Physical Medicine and Rehabilitation, California Northstate University College of Medicine, Elk Grove, USA; 2 Internal Medicine, California Northstate University College of Medicine, Elk Grove, USA; 3 Psychiatry, California Northstate University College of Medicine, Elk Grove, USA; 4 Medicine, California Northstate University College of Medicine, Elk Grove, USA; 5 Emergency Medicine, California Northstate University College of Medicine, Elk Grove, USA; 6 Surgery, California Northstate University College of Medicine, Elk Grove, USA; 7 Pain, California Center of Pain Medicine and Rehabilitation, Fair Oaks, USA

**Keywords:** ankylosing spondylitis, diagnosis, elastography, review, shear wave elastography

## Abstract

Ankylosing spondylitis (AS) is an autoimmune disease characterized by inflammation of the axial skeleton, sacroiliac (SI) joint, and entheses and typically presents with back pain/stiffness, disability, and decreased function. Early diagnosis is essential in improving the clinical outcomes of this disease. This review aims to evaluate the mechanical action of shear wave elastography (SWE) and its potential role in diagnosing and monitoring the treatment of AS. SWE is a newer ultrasound technique that utilizes low-frequency vibrations to assess tissue stiffness. Therefore, due to the progressive stiffness seen in AS, elastography can potentially evaluate and measure treatment response in AS by assessing tissue composition and elasticity. Strain elastography (SE), conversely, relies on externally applied compressive force to indirectly measure tissue stiffness by measuring the amount of tissue displaced. However, diagnostic value is limited by the ability of the operator to apply consistent force and provide absolute elasticity measurements. SWE and SE have been used to detect early musculoskeletal changes commonly seen in AS and offer advantages over conventional imaging modalities that may not be as sensitive. SWE has shown effectiveness through its quantitative measurement of tissue stiffness, which offers potential for better monitoring of disease progression and treatment effectiveness.

## Introduction and background

Ankylosing spondylitis (AS) is a progressive systemic autoimmune disease characterized by chronic inflammation of the axial skeleton, sacroiliac (SI) joint, peripheral joints, digits, and entheses. Patients typically present with back pain and stiffness, disability, and decreased function [1]. Given these symptoms, AS can lead to impaired physical fitness and subsequently affect emotional well-being. Thus, early diagnosis is important in improving the clinical outcomes of AS, typically via inflammatory markers and imaging using radiography, ultrasound (US), computed tomography (CT), or magnetic resonance imaging (MRI) to evaluate bone damage [2]. 

Validated imaging tools play a large role in diagnosing and monitoring AS [1]. Although conventional radiography is still used to screen for spondyloarthritis, it has low sensitivity for detecting the initial radiological changes observed in AS, as sacroilitis and other radiographic changes often appear later in the disease course [1]. Conversely, MRI, now incorporated into the Assessment of SpondylArthritis International Society (ASAS) criteria, can detect bone edema and synovitis before radiographic changes occur, but it is associated with high cost and low availability [1]. CT is also effective in visualizing structural lesions, but is limited by radiation exposure [1]. US has established roles in assessing various musculoskeletal conditions and is highly sensitive, noninvasive, and cost-effective [3,4].

A recent meta-analysis of the diagnostic value of US for AS found that US, particularly color doppler US, is a valid and reproducible technique to diagnose AS but is not very accurate [2]. However, it is important to note that US is not currently a validated standalone diagnostic tool for AS, but rather complements MRI and other radiographic techniques in practice. Nonetheless, color Doppler US was determined to be superior to power Doppler US in diagnostic accuracy and is recommended by the authors for diagnosing AS [2]. US elastography is a newer US technique that utilizes low-frequency vibrations to assess tissue stiffness in response to an internal or external force. Elastography consists of two main techniques: strain elastography (SE) and shear wave elastography (SWE) [5,6]. Although a 2020 meta-analysis found that SWE in rotator cuff tendinopathy/tears did not identify a significant difference in supraspinatus tendon elasticity, the authors reported that standardized scanning protocols and outcome measurements should be applied in future studies [5]. Thus, given the potential of SWE in assessing these musculotendinous structures and that enthesis of the rotator cuff is relatively common in early spondyloarthritis, continued exploration of SWE in this context may be warranted. Due to the progressive muscle stiffness seen in AS, elastography has the potential to evaluate and measure treatment response in AS by assessing tissue composition and elasticity. Additionally, the systemic involvement of AS may have manifestations in other musculoskeletal locations that can also be monitored using elastography [7]. 

Thus, this narrative review aims to elucidate the mechanisms and characteristics of elastography, evaluate the current literature’s findings on its potential role in diagnosing and monitoring treatment for AS, and provide a rationale for further investigation into this technique.

## Review

Ankylosing spondylitis

AS is an autoimmune disorder and a major subtype of spondyloarthropathies, a group of related inflammatory arthritic diseases sharing clinical and pathophysiological overlap distinct from rheumatoid arthritis [1]. The symptoms of AS generally appear during the second or third decade of life, with men being two to three times more likely to develop the disorder [1]. However, more recent studies have found that although men have a worse radiological prognosis, women have a higher disease burden due to different disease manifestations, longer delay in diagnosis, and decreased responsiveness to typical treatment for AS [8]. Though the etiology of AS is still incompletely understood, the disease is widely believed to result from a combination of genetic and environmental factors. Twin studies and familial risk analyses have demonstrated significant hereditary determination in the development of the disease [9]. In particular, there is a clear immunological genetic contribution to AS susceptibility with major and minor histocompatibility complex genes being implicated, most notably HLA-B27 [10]. While this genetic component is believed to be most significant in the pathogenesis of AS, other factors, including abnormalities of gut microbiota and variable cytokine expression (TNFα and IFNγ), have also been proposed to play a role [11,12]. Two relatively new nonsynonymous single nucleotide polymorphisms related to AS were also found, ERAP1 and IL23R, pointing to the need for further research and discovery of potential diagnostic markers [13,14]. 

AS is primarily a disease of the axial skeleton and related structures, often constituting a series of inflammatory clinical features (sacroiliitis, enthesitis, oligoarticular arthritis) in addition to certain extra-articular manifestations like anterior uveitis, psoriasis, and inflammatory bowel disease (IBD) [15]. Common chronic concerns include stiffness, pain, fatigue, poor sleep, self-consciousness, anxiety, and adverse effects of medication, with impairment of daily home and work activities reported in up to 20-25% of patients in one study [16]. Histological analysis of the affected tissues in AS patients has revealed early myxoid subchondral bone changes and synovitis of the SI joint, which eventually leads to the obliteration and replacement of articular regions by new bone and chondral fusion, causing ankylosis [17]. This disease process is associated with high leukocyte levels and edema of the bone marrow bordering cartilaginous areas of the joints and intervertebral discs; immune cell infiltration of these sites, namely by T-cells and macrophages, is believed to result in the inflammation, fibrosis, and bony replacement that characterize the musculoskeletal symptoms of AS [1].

Diagnosis of ankylosing spondylitis is based on clinical signs and symptoms and radiographic evidence of sacroiliitis. X-ray imaging of the pelvis from a Ferguson view, an angled cephalad view of the pelvis, allows for clearer visualization of the sacroiliac joints [18]. Sacroiliitis is graded on a scale of 0 - IV, with 0 indicating normal joint structure and IV indicating complete ankylosis of the sacroiliac joint [18]. Beyond X-ray imaging, an MRI of the sacroiliac joints may be useful for observing more subtle inflammatory changes in the joint [1,19]. Bone marrow edema may be present on T2-weighted imaging in individuals with AS, but this finding is nonspecific and may also be present in up to 23% of patients with mechanical back pain [1]. 

As mentioned before, early diagnosis of the condition for treatment can improve patient outcomes. Therefore, the SWE and SE diagnostic capabilities of AS are vital for prompt management. Different diagnostic parameters have been used and studied to determine disease progression. The New York Classification Criteria are generally used for diagnosing AS due to the lack of reliable and specific laboratory testing [18]. The criteria involve both a clinical physical exam component and a radiological component. Clinical criteria include low back pain and stiffness for more than three months that is improved by exercise, limitation of lumbar spine motion, and limitation of chest expansion compared to normal values of the same sex and age [20]. Radiological components include a grade 2 or higher for bilateral sacroiliitis or grade 3 and higher for unilateral sacroiliitis, with grade 0 being normal and grade 4 being severe abnormalities. A definite AS diagnosis is made with radiological findings plus one clinical finding. A probable AS diagnosis can be made with three clinical signs or only with radiological findings [20]. With the ASAS classification criteria, AS diagnoses require both the clinical signs and radiological findings [21]. Patients with back pain that exceeds three months with onset before 45 years of age can be classified by either 1) sacroilitis on imaging plus one spondylarthritis symptom or 2) HLA-B27 positivity plus at least two spondylarthritis symptoms [21]. Although radiological findings are essential for detecting the characteristic changes of AS, they must be contextualized with the broader clinical context, as AS-like findings can be found in other conditions such as diffuse idiopathic skeletal hyperostosis (DISH) or trauma [21]. 

In terms of laboratory findings, none are currently sufficient for the definitive diagnosis of AS. Individuals with AS are seronegative for rheumatoid factor and antinuclear antibodies and typically do not have elevations in inflammatory markers like erythrocyte sedimentation rate (ESR) and C-reactive protein (CRP) [18]. Although 90% of patients are positive for HLA-B27, AS can occur in the absence of HLA-B27, and previous studies have demonstrated the discordance between both incidence and severity of AS in twins positive for this haplotype [18]. Further, only 1-5% of individuals who are HLA-B27 positive will develop the disease; as such, HLA-B27 cannot be reliably used as a diagnostic marker for AS [1]. 

Diagnostic usage

While a greater understanding of the pathophysiology of AS is important, a major challenge is in early and accurate detection of the condition. Conventional radiography is able to detect late structural changes, while MRI, although it is highly sensitive and part of ASAS classification criteria, is limited by cost and easy availability [1,21]. CT can provide great structural detail, but it also exposes patients to radiation. US is safe and inexpensive, but its role in axial disease is not validated, though it is effective for peripheral joints and enthesitis [3,4]. SWE, on the other hand, builds upon the advantages of US but also provides quantitative measures of tissue stiffness, potentially preceding the structural changes seen on conventional imaging [5,6]. This may allow earlier detection and precise monitoring of AS musculoskeletal changes. 

Radiological diagnostic parameters are also being used to diagnose AS with further certainty. A study done by Wang et al. used SWE to Young’s modulus (YM), cross-sectional area (CSA), and thickness of the lumbar multifidus (LM) muscle in AS and non-AS cohorts [6]. It was found that YM in AS patients was significantly higher, which can be interpreted as being stiffer and more resistant to changing shape [6]. Percentage change in CSA and thickness before and after contraction was also measured, showing a lower percentage for both parameters in AS patients compared to asymptomatic patients. 

With acute phase reactants, the low sensitivity and specificity limit the degree of certainty with which AS can be diagnosed. However, Wang’s study showed an 83.33% sensitivity using specific cut-off values for YM for early diagnostic identification of AS in patients. Although specificity was 53.33%, due to these YM findings not being unique to AS patients, the sensitivity can be further increased to 90.74% when combining cut-off values for both YM and the change in thickness of the lumbar multifidus muscle [6]. Notably, US has good to high intra- and inter-rater reliability for assessing LM muscle thickness and percentage change [22,23]. This further supports the advantage of radiological findings in diagnosing AS, especially when combined with clinical or laboratory findings. Wang et al. are also conducting a clinical study using SWE to study the elasticity of lumbar paraspinal and multifidus muscles for AS patients who were undergoing acupuncture therapy, which could potentially further increase diagnostic sensitivity and specificity for AS [24]. 

Other radiological modalities and patient-reported outcomes are vital in determining disease progression and patient quality of life. Atik et al. used SWE and found a significant difference in stiffness between AS and asymptomatic groups, but also a significant negative correlation between the duration of the disease and Achilles stiffness (p<0.05 for both) [25]. In a study by Zhu et al., color Doppler ultrasonography was used to measure blood flow to the sacroiliac joint in AS patients [26]. It was found that a significant increase in venous flow signs was found in AS patients, with different venous flow patterns near the sacroiliac joint. These two studies are summarized in Table 1.

**Table 1:** Study and Patient Characteristics of Shear-Wave Elastography Studies for Ankylosing Spondylitis AS: ankylosing spondylitis; MHz: megahertz

Authors	Journal	Number of Patients	Imaging Technique	Outcomes Assessed
Atik 2024 [24]	J Clin Ultrasound	96 (48 AS, 48 control)	High-resolution linear 6-15 MHz probe	Bilateral Achilles tendon stiffness, thickness, and vascularity
Wang 2022 [23]	BMC Musculoskelet Disord	57 (30 AS, 27 control)	Supersonic MACH 20 Imaging System with linear-array transducer and 4-15 MHz bandwidth	Young’s modulus (YM), Lumbar multifidus (LM) cross-sectional area (CSA), and thickness

Another important patient-reported outcome measurement is the Ankylosing Spondylitis Quality of Life Scale, where patients can quantitatively express how their daily needs are being accomplished with the disease, and how much their current disease state is hindering their quality of life [27]. In terms of functionality, patients can report Bath Ankylosing Spondylitis Metrology Index (BASMI), Bath Ankylosing Spondylitis Disease Activity Index (BASDAI), and Bath Ankylosing Spondylitis Functional Index (BASFI), all of which can give a better understanding of disease progression and spinal mobility as perceived by the patients themselves [28]. Surveys such as the Visual Analogue Scale (VAS) for pain and Fatigue Scale-14 (FS-14) can inform others of the level and intensity of sensations the AS patients are experiencing [29,30]. All of these reported outcomes, coupled with diagnostic imaging, can give healthcare providers a better understanding of the disease progression from a physiological and subjective point of view for patients.

Elastography techniques

SE and SWE are advanced ultrasound-based imaging techniques that evaluate tissue stiffness, often altered in diseases involving fibrosis, inflammation, or structural changes [31]. In the context of musculoskeletal disorders like AS, these modalities have emerged as valuable non-invasive tools for assessing early structural changes [7]. Both techniques can help visualize early inflammatory changes and detect increased stiffness in areas commonly affected by the disease, such as the SI joints, entheses, and spine.

SE measures tissue deformation in response to an externally applied compressive force [32]. During an SE examination, the amount of tissue displacement (strain) is calculated and used as an indirect measure of tissue stiffness [33]. In stiffer tissues, such as those affected by inflammatory and fibrotic processes in AS, displacement is lower. On the other hand, healthy or more elastic tissues display greater displacement [32]. While SE provides qualitative or semi-quantitative information on stiffness, its diagnostic application is limited by its reliance on the operator to apply consistent force and its inability to provide absolute elasticity measurements [34]. In one study, strain ultrasound elastography was performed in longitudinal and transverse planes on 22 Achilles tendons of 11 AS patients [7]. This study found that strain elastography did not reveal significant differences in Achilles tendon stiffness between AS patients and controls, suggesting limited potential for using tendon stiffness alone as a diagnostic marker [7]. However, strain elastography may still be useful for monitoring AS, as it can help rule out early mechanical changes in tendon properties that could indicate disease progression or treatment response.

**Figure 1:**
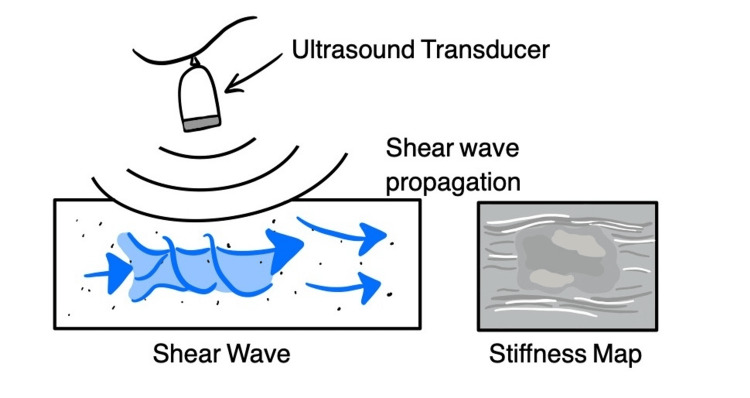
Principle of Shear-Wave Elastography Figure Credits: Megan Kou, Jimmy Wen; Tool Used: Notability

SWE is another ultrasound-based imaging technique that directly quantifies tissue stiffness by generating shear waves within the tissue using focused ultrasound pulses, which can be seen in Figure 1 [35].

These shear waves propagate through the tissue, and their speed is directly related to tissue stiffness. Faster shear wave speeds indicate stiffer tissue, while slower shear wave speeds indicate looser tissue [35]. This modality provides a quantitative, reproducible measurement of elasticity that does not rely on external compression [36]. Instead, it offers more standardized and repeatable results than SE, making it particularly valuable for serial monitoring. A case-control study explored SWE using a high-resolution linear 6-15 MHz probe in 48 AS patients and 48 asymptomatic individuals [25]. The study found that SWE revealed a significant decrease in Achilles tendon stiffness in AS patients compared to healthy controls, indicating early tendon deterioration in AS. These findings showed that tendon stiffness decreased progressively with disease duration, suggesting SWE has the potential to be utilized as a tool for both diagnosing AS and monitoring disease progression [25].

## Conclusions

SWE has shown promising potential as a sensitive and non-invasive tool to evaluate tissue stiffness in patients with AS. SWE and SE have been used to detect early musculoskeletal changes commonly seen in AS and offer advantages over conventional imaging modalities that are highly sensitive but may be less widely accessible. SWE has shown effectiveness through its quantitative measurement of tissue stiffness which offers potential for better monitoring of disease progression and treatment effectiveness. The use of SWE in clinical practice can enhance the treatment of AS. Further research is required to optimize the elastography protocols and validate their utility in a clinical setting for more diverse patient populations. Additionally, the usage of SWE can be considered in future studies to monitor the progression of the disease.
